# Tungsten Promoted Ni/Al_2_O_3_ as a Noble-Metal-Free Catalyst for the Conversion of 5-Hydroxymethylfurfural to 1-Hydroxy-2,5-Hexanedione

**DOI:** 10.3389/fchem.2022.857199

**Published:** 2022-03-09

**Authors:** Ying Duan, Rui Wang, Qihang Liu, Xuya Qin, Zuhuan Li

**Affiliations:** ^1^ College of Food and Drug, Luoyang Normal University, Luoyang, China; ^2^ Henan Key Laboratory of Function-Oriented Porous Material, College of Chemistry and Chemical Engineering, Luoyang Normal University, Luoyang, China

**Keywords:** Ni/Al_2_O_3_, HMF, hydrogenation, W promoted, hydroxy-hexanedione

## Abstract

The conversion of 5-hydroxymethylfurfural (HMF) to 1-hydroxy-2,5-hexanedione (HHD) represented a typical route for high-value utilization of biomass. However, this reaction was often catalyzed by the noble metal catalyst. In this manuscript, W promoted Ni/Al_2_O_3_ was prepared as a noble-metal-free catalyst for this transformation. The catalysts were characterized by XRD, XPS, NH_3_-TPD, TEM, and EDS-mapping to study the influence of the introduction of W. There was an interaction between Ni and W, and strong acid sites were introduced by the addition of W. The W promoted Ni/Al_2_O_3_ showed good selectivity to HHD when used as a catalyst for the hydrogenation of HMF in water. The influences of the content of W, temperature, H_2_ pressure, reaction time, and acetic acid (AcOH) were studied. NiWOx/Al_2_O_3_-0.5 (mole ratio of W:Ni = 0.5) was found to be the most suitable catalyst. The high selectivity to HHD was ascribed to the acid sites introduced by W. This was proved by the fact that the selectivity to HHD was increased a lot when AcOH was added just using Ni/Al_2_O_3_ as catalysts. 59% yield of HHD was achieved on NiWOx/Al_2_O_3_-0.5 at 393 K, 4 MPa H_2_ reacting for 6 h, which was comparable to the noble metal catalyst, showing the potential application in the production of HHD from HMF.

## Introduction

The production of chemicals from a renewable resource is one of the essential tasks for sustainable chemistry (Corma et al., 2007; Besson et al., 2014; Mika et al., 2017; Fang et al., 2020; Xu et al., 2020). As the 5-hydroxymethylfurfural (HMF) could be obtained easily by the dehydration of hexoses (Yu and Tsang, 2017; Fan et al., 2019; Kang et al., 2019; Chang et al., 2021; Das and Mohanty, 2021; Guo et al., 2021; Tempelman et al., 2021), a class of compounds abundant in nature, the transformation of HMF is one of the hot topics for sustainable chemistry (Averochkin et al., 2021; Bielski and Grynkiewicz, 2021; Fang and Riisager, 2021). Many studies had focused on the conversion of HMF to various products with potential or practice applications. It was reported that fuels (Esteves et al., 2020) and their additives (Nagpure et al., 2020), polymer monomers (Duan et al., 2017a; Elsayed et al., 2020; Wang et al., 2020; Fulignati et al., 2021) and other chemicals (Ohyama et al., 2017; Ramos et al., 2017; Wozniak et al., 2019; Zhang et al., 2019) with the high added value could be produced using HMF as feedstock through catalytic hydrogenation (Ohyama et al., 2016; Ren et al., 2016; Yang et al., 2019a; Long et al., 2019; Han et al., 2020; Wiesfeld et al., 2020; Gao et al., 2021), oxidation (Neatu et al., 2016; Martínez-Vargas et al., 2017; Deshan et al., 2020), etherification (Che et al., 2015) and other catalytic procedures (Karve et al., 2020; Zhang et al., 2021).

1-hydroxy-2,5-hexanedione (HHD) was one of the high value-added compounds obtained from HMF through catalytic hydrogenation (Schiavo et al., 1991; Gupta et al., 2015; Zhu et al., 2019; Yang et al., 2020). The HHD could be used for the preparation of polyols, nitrogen and oxygen heterocycles. Recently, it was reported that the HHD could convert to 2-hydroxy-3-methyl-2-cyclopenten-1-one (MCP) through an intramolecular aldol condensation procedure at mild conditions (Duan et al., 2017b; Wozniak et al., 2018). The MCP was a commercialized edible essence produced from petrochemical feedstock by multistep reactions with low yield and severe pollution. The HHD was a potential feedstock candidate for the improvement in the production of MCP.

The transformation of HMF to HHD had been reported by several groups. The reaction was usually conducted in water under H_2_ pressure. The formic acid could also be used as the hydrogen source when a homogeneous catalyst was used (Xu et al., 2017). Acid additives such as HCl, Amberlyst-15 or H_3_PO_4_ was usually necessary for this conversion (Liu et al., 2014). To avoid acid additives, the introduction of acid sites in the catalyst was a good choice. For example, the reaction could conduct without the addition of acid additives when acid support MIL-101 (Yang et al., 2019b), zeolite (Ramos et al., 2019) or Nb_2_O_5_ (Duan et al., 2017b) was used for the preparation of Pd catalyst. The supported Pd was found to be an effective heterogeneous catalyst for this transformation while the Ir complexes were a good homogeneous catalyst candidate (Xu et al., 2017). The Ru (Gupta et al., 2015) complex and supported Au (Ohyama et al., 2014) could also catalyse this reaction. The employment of noble-metal was a barrier for the further application of the conversion of HMF to HHD. To reduce the use of noble-metal, high-performance catalyst with low noble-metal load, high dispersion and activity was designed and applied for this transformation.

The application of noble-metal-free catalyst for the conversion of HMF to HHD without acid additives was a better choice for the improvement. However, there was only one report that HMF in water (∼0.3 wt%) could be converted to HHD by Ni_2_P nanoparticles up to now (Fujita et al., 2020). In this manuscript, the tungsten promoted Ni/Al_2_O_3_ was simply prepared and used as a noble-metal-free catalyst for the transformation of HMF to HHD. The catalyst showed high activity and stability in the reaction. The introduction of tungsten in the catalyst improved the activity and selectivity to HHD greatly.

## Results and Discussion

### Characterization

**FIGURE 1:**
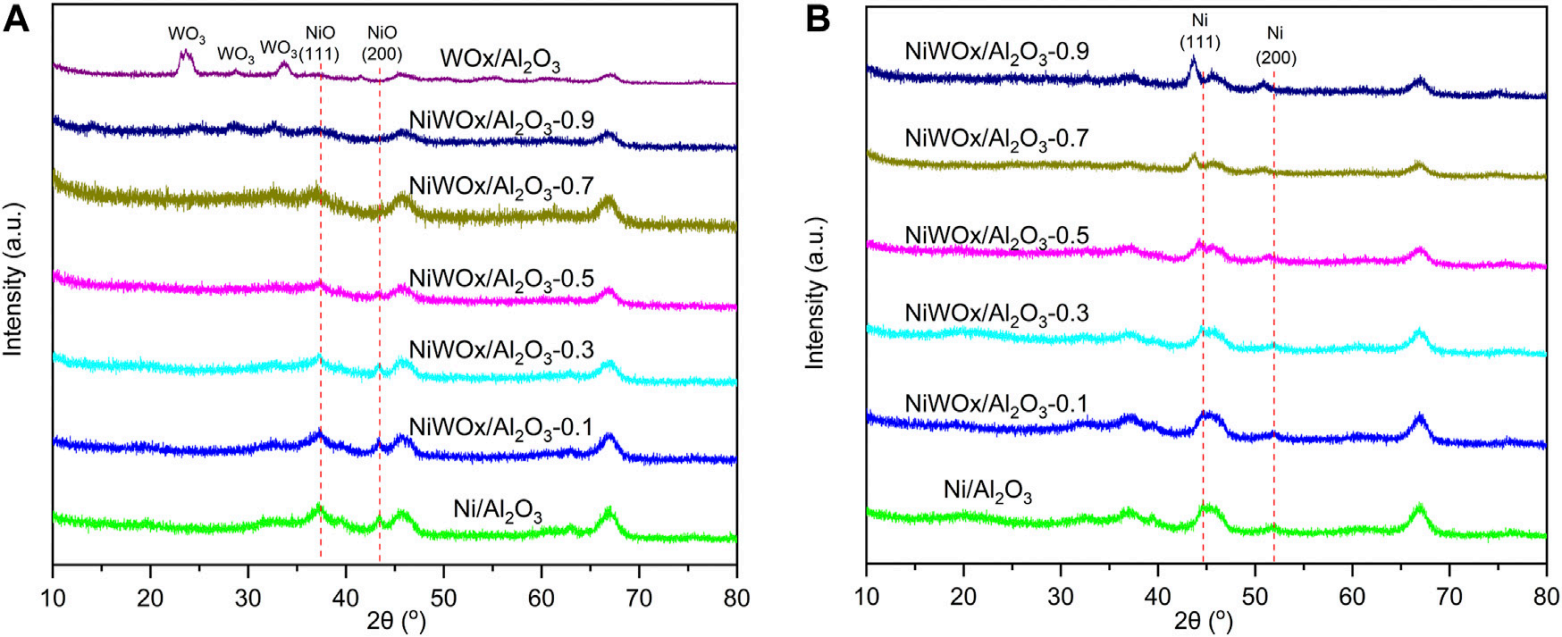
The XRD patterns of NiWOx/Al_2_O_3_ with different content of W before **(A)** and after **(B)** H_2_ reduction.

The X-ray diffraction (XRD) patterns of NiWOx/Al_2_O_3_ with different content of W was displayed in Figure 1. Figure 1A were the results of samples after calcined. Except for the diffraction peaks for *γ*-Al_2_O_3_, Ni/Al_2_O_3_ had additional two peaks at 37.3^o^ and 43.4^o^. This should be ascribed to (111) and (200) diffraction peaks of NiO (PDF#47-1049). The two peaks decreased as the content of W increased for NiWOx/Al_2_O_3_ and almost disappeared for NiWOx/Al_2_O_3_-0.7 and NiWOx/Al_2_O_3_-0.9. The WOx/Al_2_O_3_ showed (001) (020) (200) (111) (021) (201), and (220) peaks of WO_3_ without NiO peaks (Supplementary Figure S1). W species mainly existed as WO_3_ after the calcination. However, no diffraction peaks ascribed to WOx could be found for NiWOx/Al_2_O_3_ showed that the W had a reasonable degree of dispersion on the support. After the Ni/Al_2_O_3_ was reduced in H_2_, the diffraction peaks ascribed to NiO disappeared (Figure 1B). Two new peaks corresponding to Ni (111) and (200) were immersed at 44.5^o^ and 51.9^o^ (PDF#04-0850). This showed that most of the NiO could be reduced to Ni by H_2_ reduction, which was consistent with the results of H_2_-Temperature programmed reduction (H_2_-TPR) (Supplementary Figure S2 and Table S1). After the introduction of W, NiWOx/Al_2_O_3_ still had the diffraction peaks of Ni. The diffraction peaks moved to a smaller angle with increased W content. This should be caused by the W atoms entering the lattice of Ni.

**FIGURE 2:**
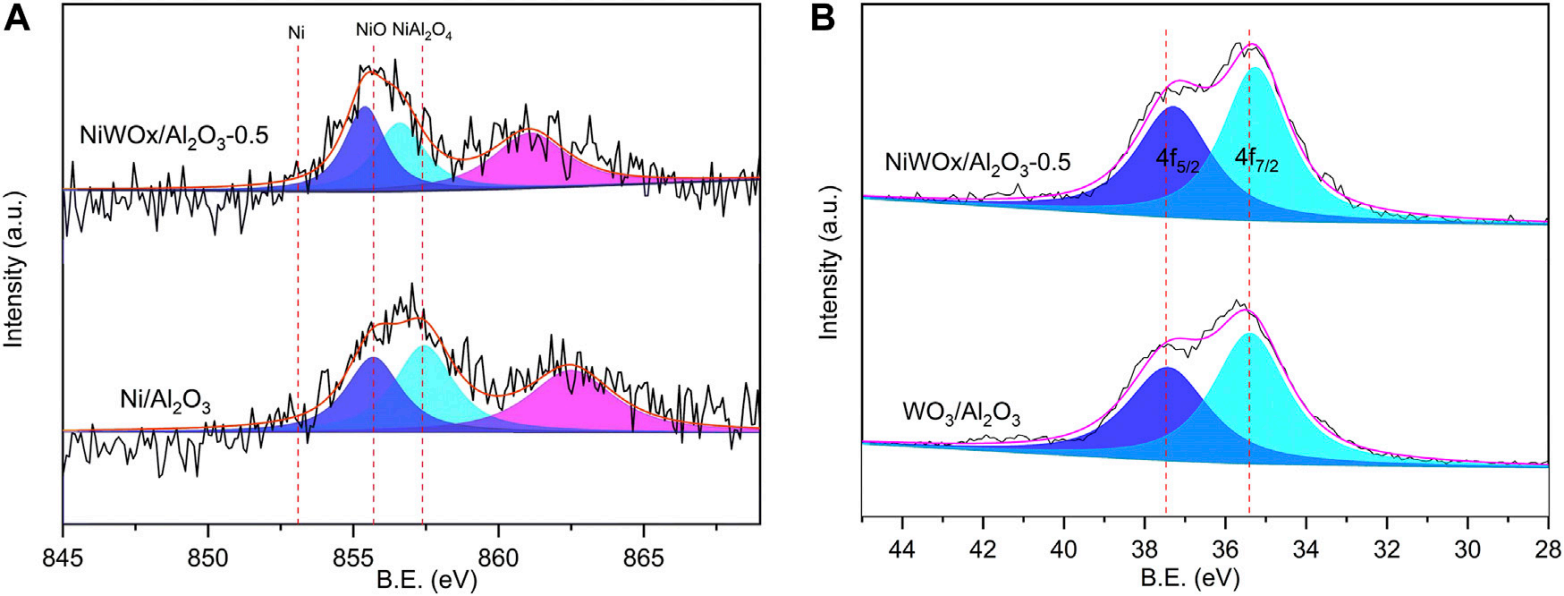
The XPS spectra of Ni 2p **(A)** and W 4f **(B)**.

The surface chemical state was characterized by X-ray photoelectron spectroscopy (XPS) and the results were shown in Figure 2 and Supplementary Figure S3. After deconvolution operation, the Ni/Al_2_O_3_ showed two peaks centered at 856.7 and 855.3 eV, which should be ascribed to NiAl_2_O_4_ and NiO, respectively (Figure 2A) (Yang et al., 2016). No peaks (853.0 eV) ascribed to metallic Ni could be found in the XPS spectra. This should be caused by the oxidation of surface Ni when exposed to air. The binding energy shifted to higher energy when W was introduced. The binding energy peaks of NiAl_2_O_4_ and NiO were 857.4 and 855.8 eV, respectively for NiWOx/Al_2_O_3_-0.5. This decrease indicated that the W had interaction with Ni. This interaction between Ni and W should weaken that between Ni and Al. Consequently, the relative content of NiAl_2_O_4_ decreased after the introduction of W revealed by the result of XPS (Figure 2A). The interaction between Ni and W was further proved by the XPS spectra of W (Figure 2B). The WOx/Al_2_O_3_ had peaks at 37.4 and 35.4 eV corresponding to the binding energy of WO_3_ of 4f_5/2_ and 4f_7/2_ (Cao et al., 2014). This value decreased to 37.3 and 35.3 for NiWOx/Al_2_O_3_-0.5. The interaction between Ni and W was also consistent with the results of H_2_-TPR that the reduction temperature peak was changed after the introduction of W (Supplementary Figure S2; Table S1).

**FIGURE 3:**
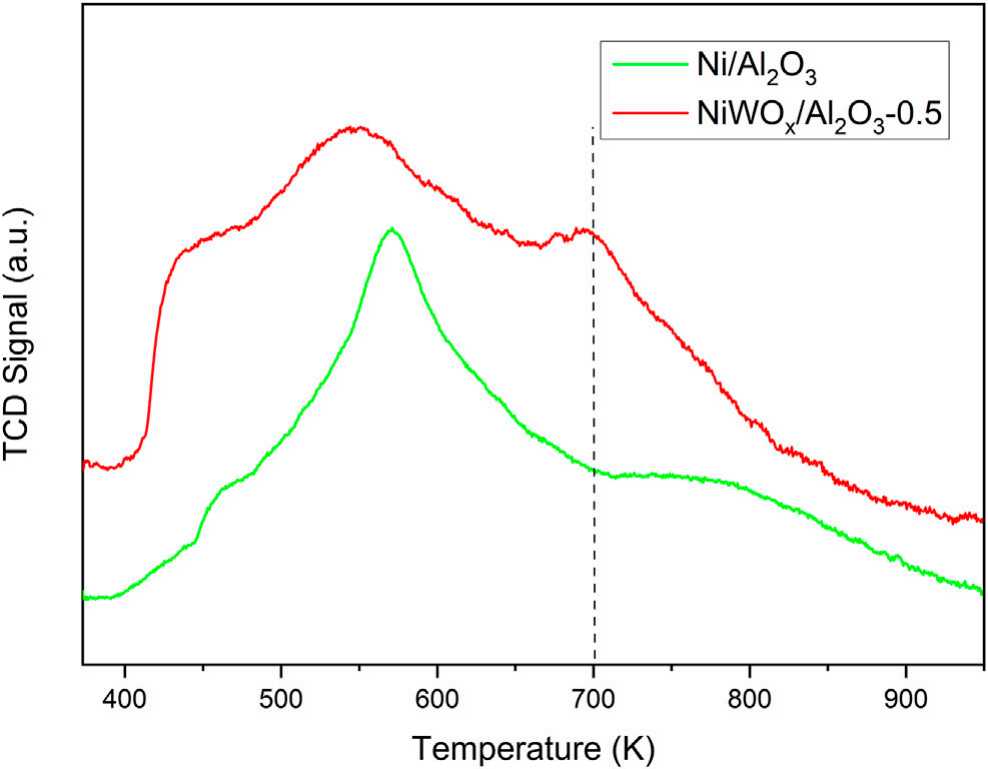
The NH_3_-TPD profiles of Ni/Al_2_O_3_ and NiWOx/Al_2_O_3_-0.5.

The acidic properties of Ni/Al_2_O_3_, NiWOx/Al_2_O_3_-0.5 and WOx/Al_2_O_3_ were investigated by the temperature-programmed desorption of ammonia (NH_3_-TPD), and the results were shown in Figure 3 and Supplementary Figure S4. The Ni/Al_2_O_3_ showed a sharp peak at 570 K which was ascribed to the weak to medium acid site of Al_2_O_3_. The NH_3_-TPD of NiWOx/Al_2_O_3_-0.5 showed three wide peaks centered at 442, 544, and 700 K corresponding to the weak acid, medium acid, and strong acid sites respectively. The NiWOx/Al_2_O_3_-0.5 posed a relatively strong acid site except for the weak to medium acid site compared to Ni/Al_2_O_3_. This desorption curve was similar to WOx/Al_2_O_3_ (Supplementary Figure S4). Hence, the strong acid site was produced due to the introduction of W in NiWOx/Al_2_O_3_-0.5.

**FIGURE 4:**
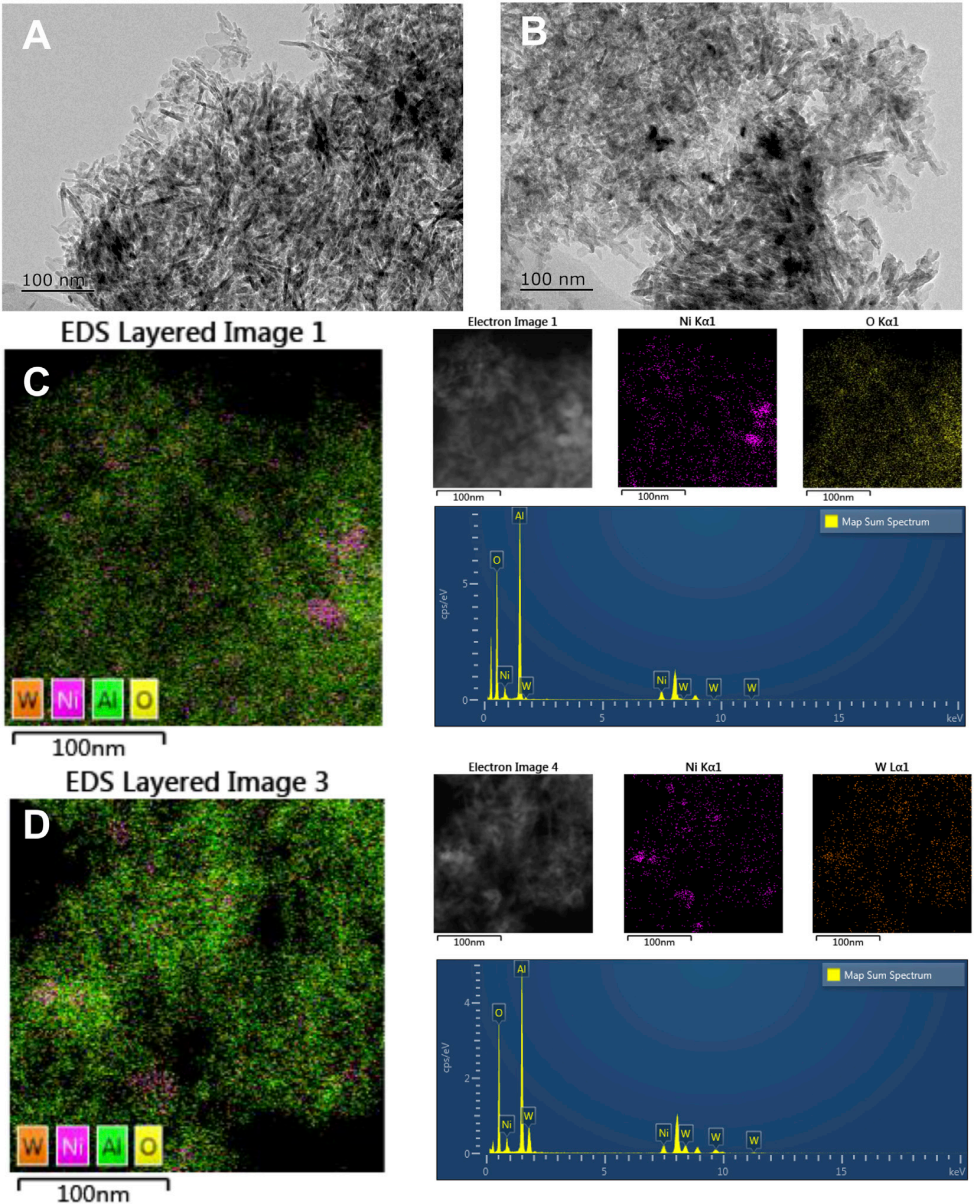
The TEM images and EDS mappings of Ni/Al_2_O_3_ **(A,C)** and NiWOx/Al_2_O_3_-0.5 **(B,D)**.

The morphology of Ni/Al_2_O_3_ and NiWO_x_/Al_2_O_3_-0.5 was characterized by transmission electron microscopy (TEM). The TEM images (Figure 4) showed that the Ni was distributed on the surface of the support in granular or rod form for both Ni/Al_2_O_3_, NiWOx/Al_2_O_3_-0.5 in nanoscale. To get a clearer distribution of elements, the energy-dispersive spectrometer (EDS) mapping was taken for both catalysts. The results were shown in Figures 4C,D, Supplementary Figures S5–8 and Supplementary Table S2. The Supplementary Table S2 summarized the percentage of each element in Ni/Al_2_O_3_ and NiWOx/Al_2_O_3_-0.5. The content of Ni, W was close to the theoretical value. It could be seen that the distribution of Ni could be divided into two categories for Ni/Al_2_O_3_. Some of the Ni gathered together to form metal particles as shown in TEM images. While the other Ni did not agglomerate and dispersed on the support evenly. This should be ascribed to the unreduced Ni species with high interaction between Al. After the introduction of W, the NiWOx/Al_2_O_3_-0.5 had a similar Ni distribution as Ni/Al_2_O_3_ (Figure 4D). The EDS mappings of W showed that the Ni and W did not have a separated distribution. The W always distributed followed the distribution trend of Ni. This was advantageous for the synergistic effect between Ni and WOx.

### Hydrogenation of HMF

**FIGURE 5:**
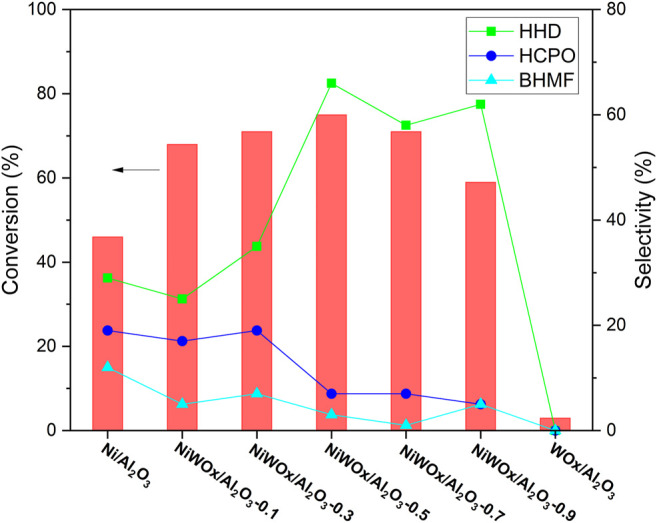
The hydrogenation of HMF on different catalyst. Reaction conditions: catalysts (20.0 mg), HMF solution (2.00 g, HMF: 1 mmol), H_2_ (4 MPa), 413 K, 2 h.

The hydrogenation of HMF was conducted in water under a hydrogen atmosphere. We first checked the effect of W for the reaction, and the results were shown in Figure 5. 46% conversion of HMF was obtained when Ni/Al_2_O_3_ without W was used as the catalyst. HHD was detected as one of the products, proving that the reaction could be catalyzed by Ni catalyst. However, the selectivity to HHD was only 29% accompanied by 19% selectivity to 3-(hydroxymethyl)cyclopentan-1-one (HCPO) and 12% selectivity to 2,5-Bis(hydroxymethyl) furan (BHMF). Deep hydrogenation products reported in the literature (Yao et al., 2013; Pomeroy et al., 2021; Zhang et al., 2022) for Ni and Ru based catalysts (Ni–Ce/Al_2_O_3_, Ni-Co-Al mixed oxide, and Ru/C) such as 2,5-bis(hydroxymethyl) tetrahydrofuran, 1,2,6-hexanetriol and 1,2,5-hexanetriol were not found, showing that the catalyst had moderate hydrogenation activity. When a small amount of W was introduced to the Ni/Al_2_O_3_, the conversion of HMF increased to 68% while the selectivity to HHD decreased a little to 25% for NiWOx/Al_2_O_3_-0.1. A small amount of W had no promoting effect for the selectivity to HHD. However, as the amount of W increased, the selectivity to HHD had a sharp increase to 66% for NiWOx/Al_2_O_3_-0.5 with a little increase in the conversion of HMF. When continued to increase the content of W, the selectivity to HHD maintained around 60% while the conversion of HMF decreased to 59%. An appropriate amount of W was needed for the high performance for hydrogenation of HMF to HHD. When no Ni was used in the catalyst (WOx/Al_2_O_3_), the HMF was almost unchanged showed that the W did not have the function for hydrogenation. The increased activity for the conversion of HMF could be caused by the interaction between Ni and W which was clued by the XRD, XPS, and H_2_-TPR. Firstly, the electronic state of Ni was changed by the addition of W. Secondly, the interaction between Ni and W weaken that between Ni and Al. So, the content of NiAlO_4_, a species that had no hydrogenation activity, decreased. Therefore, the hydrogenation activity increased after the introduction of W. The high selectivity to HHD should be ascribed to the strong acid site introduced by the addition of W. It was reported that the acids play a key role in the isomerization of furan rings in the conversion of HMF to HHD. The introduced strong acid sites by W were conducive to the rearrangement of furan rings during the reaction. Thus, the selectivity to HHD was enhanced.

**FIGURE 6:**
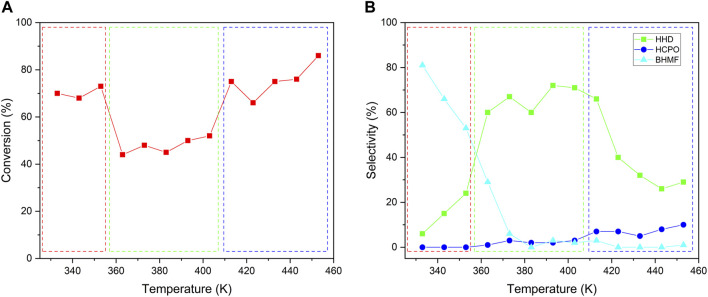
The effect of temperature on the conversion **(A)** and selectivity **(B)** for HMF conversion. Reaction conditions: NiWOx/Al_2_O_3_-0.5 (20.0 mg), HMF solution (2.00 g, HMF: 1 mmol), H_2_ (4 MPa), 333-453 K, 2 h.

The hydrogenation of HMF to HHD was a multistep reaction including hydrogenation and isomerization. The temperature was very important for this multistep reaction. We studied the effect of temperature by conducting the reaction at a temperature between 333 and 453 K and the results were shown in Figure 6. Generally, the conversion should be increased with the rise in temperature. However, the conversion of HMF experienced a process of falling first and then rising. As shown in Figure 6A, the temperature range was divided into three distinct parts. In each temperature range, the conversion of HMF increased smoothly with the rise in temperature. However, there had a sharp descent in the conversion when the temperature increased from 353 to 363 K and a sharp ascend in the conversion when the temperature increased from 403 to 413 K. We first checked the change of catalysts after reacting at different temperature by XRD (Supplementary Figure S9). It could be seen that, both the NiWOx/Al_2_O_3_-0.5 and Al_2_O_3_ had no obvious change after reacting at different temperature. This showed the catalysts was stable at the reaction condition. The changes in conversion could be interpreted by the different reaction pathways revealed by the change in selectivity (Figure 6B). At a temperature lower than 363 K, the main product was BHMF which was the hydrogenation of aldehyde in HMF. Both the selectivity to HHD and HCPO, which should be produced by the isomerization of furan rings, was very low. As the reaction temperature raised from 333 to 363 K, the selectivity to BHMF decreased while the selectivity to HHD increased. The maximum increase in the selectivity to HHD was observed when the temperature increased from 353 to 363 K. The hydrogenation active center only required the hydrogenation of aldehyde group for BHMF. However, both the aldehyde group and intermediates needed to be hydrogenated by the hydrogenation active center for the production of HHD. As a result, there had a decline in conversion when the temperature increased from 353 to 363 K. A similar phenomenon also occurred at temperatures increased from 403 to 413 K. The selectivity to HHD decreased sharply when the temperatures increased from 403 to 413 K. At the same time, the selectivity to HCPO began to increase. In a word, the hydrogenation active center should play the role of hydrogenation function in multiple steps that led to the low conversion in the transformation of HMF to HHD. The suitable temperature range for high selectivity to HHD was from 363 to 413 K.

**FIGURE 7:**
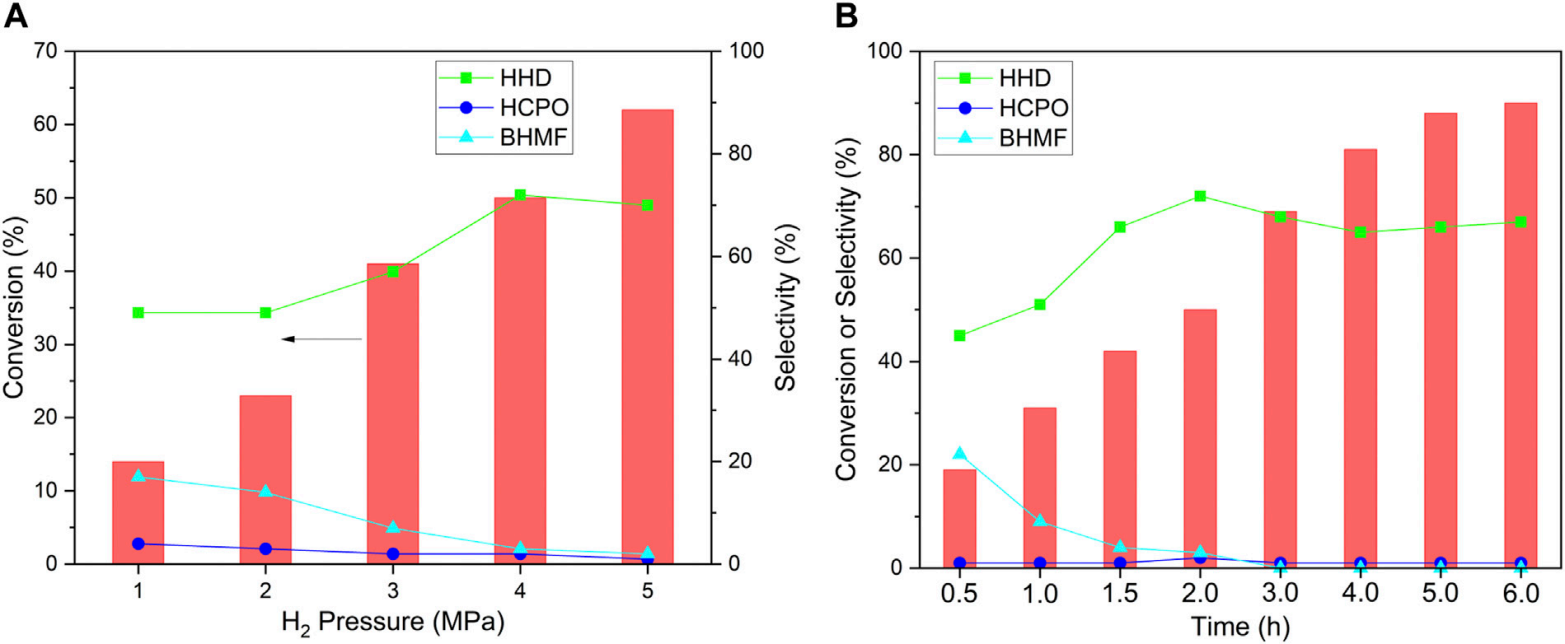
The effect of H_2_ pressure **(A)** and reaction time **(B)** for the hydrogenation of HMF. Reaction conditions: NiWOx/Al_2_O_3_-0.5 (20.0 mg), HMF solution (2.00 g, HMF: 1 mmol), H_2_ (1-5 MPa), 393 K, 0.5-6.0 h.

The effect of H_2_ pressure and reaction time was studied to optimize the reaction conditions. The conversion of HMF increased with the increase in H_2_ pressure (Figure 7). When the H_2_ pressure was lower than 4 MPa, the selectivity increased with the increase in pressure. The highest selectivity to HHD was achieved at 4 MPa H_2_. The conversion increased with the extension of time (Figure 7B). When the reaction was conducted at a shorter time, there had a low selectivity to HHD and BHMF, the intermediate for HHD, was found as the main products. However, as the reaction prolonged to 2 h, the selectivity to HHD increased to 72% while that to BHMF decreased to 3%. The selectivity to HHD kept around 65% when further extension of time. The highest yield of HHD was 59% which was obtained after 6 h reaction.

To verify the effect of acid for the conversion of HMF to HHD, the Ni/Al_2_O_3_ was used as the catalyst for this transformation with different amounts of acetic acid (AcOH). The results were shown in Table 1. As mentioned above, the selectivity to HHD was 29% without the addition of AcOH. The byproduct or intermediate were HCPO and BHMF. This result was in accordance with the previous report (Perret et al., 2016) that the HHD was one of the products when nickel/alumina was used for the hydrogenation of HMF in water. However, the selectivity to HHD was typically low (less than 15%) when no acid was used. When 10 mg of AcOH was added, there had a little decline in the conversion of HMF. However, the selectivity to HHD increased from 29 to 59% (Table 1, Entry 2). This showed that the acid was conducive to improving the selectivity to HHD rather than the conversion of HMF. Further to increase the amount of acetic acid, there was no significant further improvement in the selectivity to HHD (Table 1, Entries 3-6). The highest selectivity for HHD was 61% which was acquired with 40 mg of AcOH. These experiments proved the role of acid for the high selectivity to HHD.

**TABLE 1:** Effect of AcOH on the conversion of HMF to HHD catalyzed by Ni/Al_2_O_3_.

Entry	AcOH (mg)	Conversion (%)	Selectivity (%)		
			HHD	HCPO	BHMF
1	0	46	29	19	12
2	10	40	59	2	2
3	20	42	52	3	1
4	30	39	58	1	3
5	40	45	61	1	3
6	50	59	56	2	3

Reaction conditions: Ni/Al_2_O_3_ (20.0 mg), HMF solution (2.00 g, HMF: 1 mmol), AcOH, H2 (4 MPa), 413 K, 2.

**FIGURE 8:**
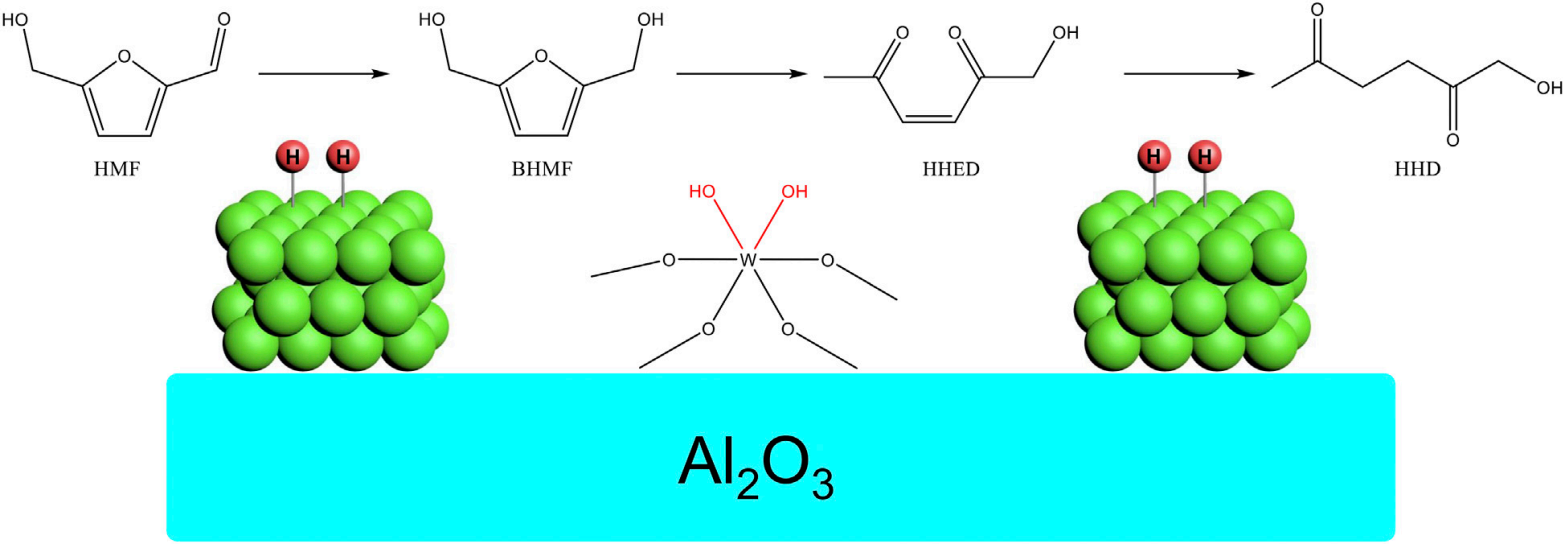
The proposed reaction pathway on NiWOx/Al_2_O_3_.

Based on the characterization and the hydrogenation of HMF, the reaction pathway and the role of catalyst was proposed as shown in Figure 8. The HMF was firstly hydrogenated to BHMF on Ni. Based on the literature and our previous work (Duan et al., 2017b; Martínez-Vargas et al., 2017; Ramos et al., 2017; Fujita et al., 2020), the BHMF was ready to isomerize to 1-hydroxyhex-3-ene-2,5-dione (HHED) catalyzed by acid. This was the crux for the reaction. In this work, the WOx played the role of acid to catalyze this transformation. At last, the HHED was hydrogenated to HHD on Ni. The suitable acidity of WOx and the moderate hydrogenation activity was the key for this multistep tandem reaction. The adjacent distribution of Ni and WOx accelerated the conversion of intermediates thus avoiding possible polymerization side reactions.

## Conclusion

In conclusion, the conversion of HMF to HHD was achieved by noble-metal-free W promoted Ni/Al_2_O_3_. The Ni and W uniformly dispersed on the surface of the support. The interaction between W and Ni increased the activity of Ni/Al_2_O_3_ for the hydrogenation of HMF. The introduction of W generated strong acid sites, which were the key for the high selectivity to HHD. The role of acid was proved by the addition of AcOH to unpromoted Ni/Al_2_O_3_. A suitable temperature was needed for the transformation of HMF to HHD smoothly. After the optimization of the conditions, a 59% yield of HHD was acquired at 393 K, 4 MPa H_2_ reacted for 6 h on NiWOx/Al_2_O_3_-0.5. This work provided the idea for high selectivity to HHD from HMF by the introduction of suitable acid and improved the feasibility of putting this reaction into practical application by using a non-noble metal catalyst.

## Materials and Method

### Materials

HMF (98%, C_6_H_6_O_3_) was bought from Zhengzhou Alpha Chemical Co. Ltd. Aluminum oxide (99.99% metals basis, ≤20 nm, Crystal form: *γ*-Al_2_O_3_), n-decane (99.8%, C_10_H_22_) and ammonium metatungstate [99.5% metals basis (NH_4_)_6_H_2_W_12_O_40_·xH_2_O] were purchased from Aladdin Chemistry Co. Ltd. Nickel (II) nitrate hexahydrate [98%, Ni(NO_3_)_2_·6H_2_O] and AcOH (99.5%, C_2_H_4_O_2_) was got from Anhui Zesheng Technology Co., Ltd.

### Characterization

A Rigaku D/Max 2500/PC powder diffractometer was used to collect the X-ray diffraction (XRD) patterns. Cu Kα radiation at 40 kV was used as the X-ray source. Thermo Escalab 250Xi spectrometer with Al Kα was used to characterize the X-ray photoelectron spectroscopy (XPS) spectra. The sample powder was overspread on a double-faced adhesive tape on aluminum foil. The sample was pressurized to 8 MPa for 30 s and used for measurement. Before measurement, the chamber pressure was vacuumized to <1 × 10^−10^ mBar. The binding energy (BE) was adjusted by the binding energy of C1s. The transmission electron microscopy (TEM) images and energy-dispersive spectrometer (EDS) elemental mappings were taken on a JEOL JEM-2100 F field emission transmission electron equipped with An Oxford 80T detector. The temperature-programmed desorption of ammonia (NH_3_-TPD) and H_2_-Temperature programmed reduction (H_2_-TPR) was conducted on the Micromeritics AutoChem II 2920 Instrument. Typically for NH_3_-TPD, 60.0 mg of sample were loaded into the sample tube. The sample was heated to 773 K under He flow (10 mL/min) and kept for 2 h. The tube was cooled to 373 K and 10NH_3_-He (30 mL/min) was introduced for 0.5 h. Then the atmosphere was switched to He (10 mL/min) and kept for 1 h to remove the physical adsorbed NH_3_. After that, the temperature was increased (10 K/min) from 373 to 973 K in an atmosphere of He (10 mL/min). The desorbed NH_3_ was detected by the thermal conductivity detector (TCD). For H_2_-TPR, the calcinated catalyst (85.0 mg) was degassed at 573 K under an atmosphere of Ar (10 mL/min) for 2 h. The sample was cooled to 373 K. The temperature was increased (10 K/min) from 373 K to 973 k under the atmosphere of 10H_2_-Ar (30 mL/min). The H_2_ consumption was monitored by a TCD detector.

### Preparation of Catalysts

In a typical procedure, Ni(NO_3_)_2_·6H_2_O (2.50 g) and (NH_4_)_6_H_2_W_12_O_40_·xH_2_O (1.05 g) were dissolved in water (10.00 g). Then Al_2_O_3_ was added to the solution. The mixture was stirred evenly to form a paste and kept standing for 24 h. Then, the paste was dried at 393 k overnight. Followed by calcined at 823 K in the air for 4 h. The obtained solid was ground to pass through 100 mesh sieve and reduced at 773 K in H_2_ to afford the NiWOx/Al_2_O_3_-0.5. The 0.5 referred to the mole ratio of W to Ni.

### Catalytic Hydrogenation

The hydrogenation reaction was conducted in a 20 mL stainless steel reactor. Typically, the HMF aqueous solution (2.00 g, HMF: 126.0 mg), catalyst (20.0 mg), and magneton were put into a glass lining. The lining was set in the reactor and sealed and purged with H_2_ for 4 times to displace the air. Then the reactor was filled with H_2_ at a specified pressure and put in an oil bath set at a certain temperature. After the reaction, 0.5 mL ethanol solution of the internal standard (n-decane) was added and the mixture was diluted to 10 mL by ethanol. After centrifugation, the liquid was used for analysis. The qualitative analysis was conducted by GC on a Shimadzu GC-2014 equipped with a SH-Rtx-1701 column (30 m × 0.32 mm × 0.25 µm). The oven temperature was started from 353 K for 2 min and raised to 523 K with 20 K/min heating rate. The oven was kept at 523 K for 1.5 min. The GC-MS was performed on Shimadzu GC/MS-TQ8040 equipped with an SH-Rxi-5Sil MS column (30 m × 0.25 mm × 0.25 µm). The oven temperature was started from 323 K for 1 min and raised to 473 K with 40 K/min heating rate and then raised to 553 K and kept at the temperature for 5 min.

## Data Availability

The original contributions presented in the study are included in the article/supplementary material, further inquiries can be directed to the corresponding author.
