# Supplementary material for: Tungsten Promoted Ni/Al2O3 as a Noble-Metal-Free Catalyst for the Conversion of 5-Hydroxymethylfurfural to 1-Hydroxy-2,5-Hexanedione
Source: Front Chem. 2022 Mar 9;10:857199. doi: 10.3389/fchem.2022.857199 (PMC8959628; doi:10.3389/fchem.2022.857199)
Supplement: Supplementary file 1 [file DataSheet1.PDF]

Supplementary Material

1 Supplementary Figures

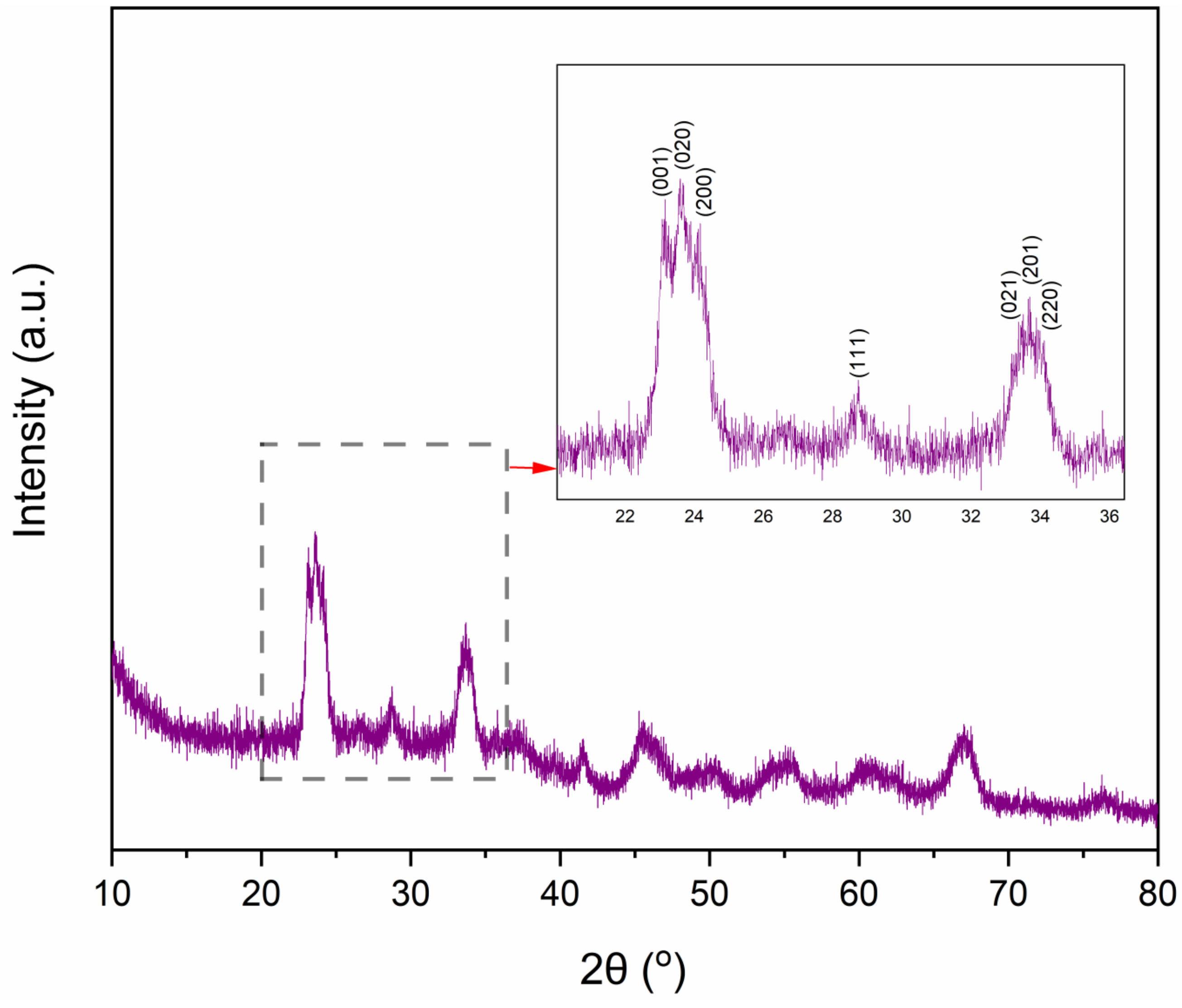

Supplementary Figure S1. The XRD pattern of WOx/Al_2O_3.

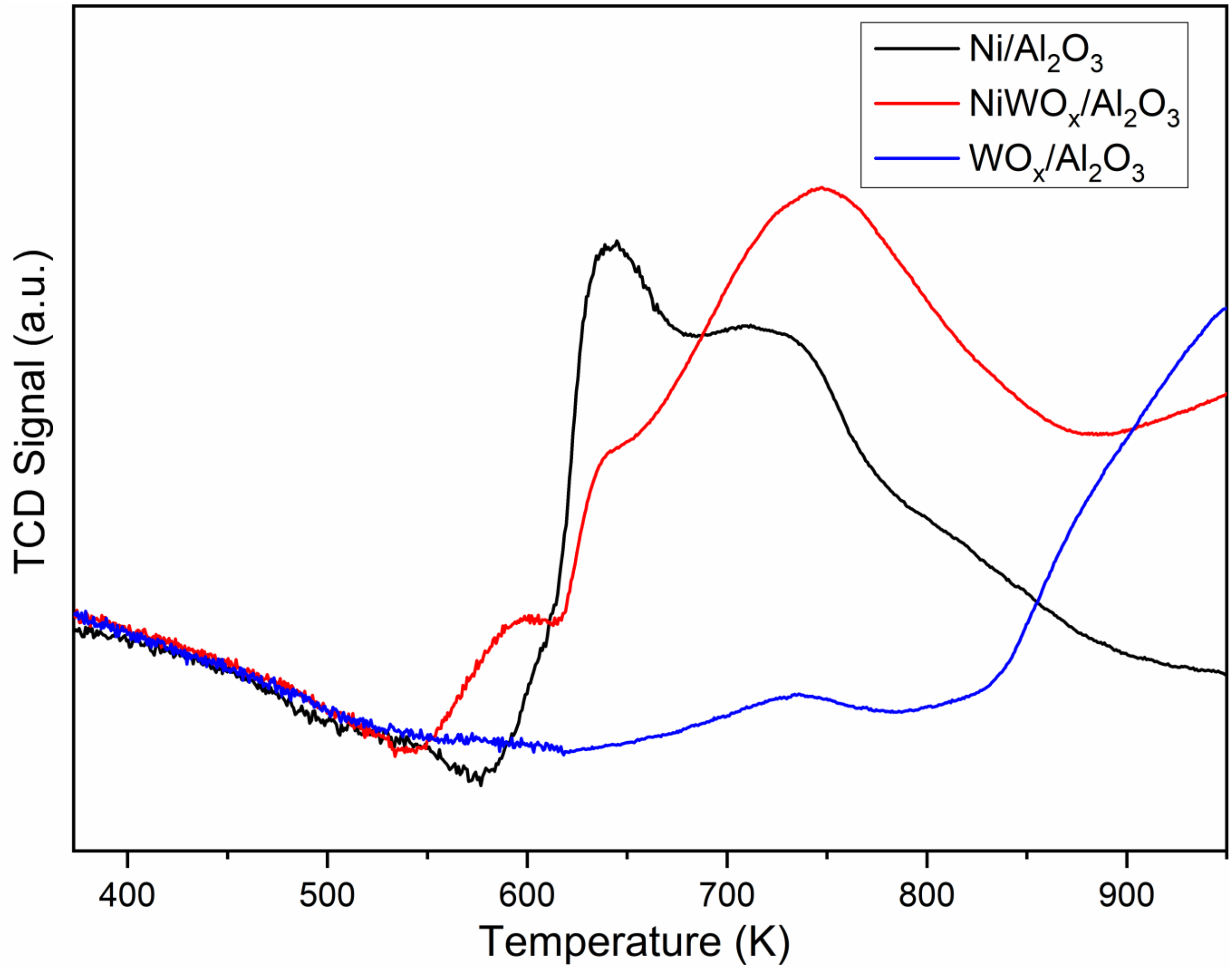

Supplementary Figure S2. The H_2-TPR profiles of Ni/Al_2O_3, $NiWOx/Al_2O_3$-0.5, and WOx/Al_2O_3.

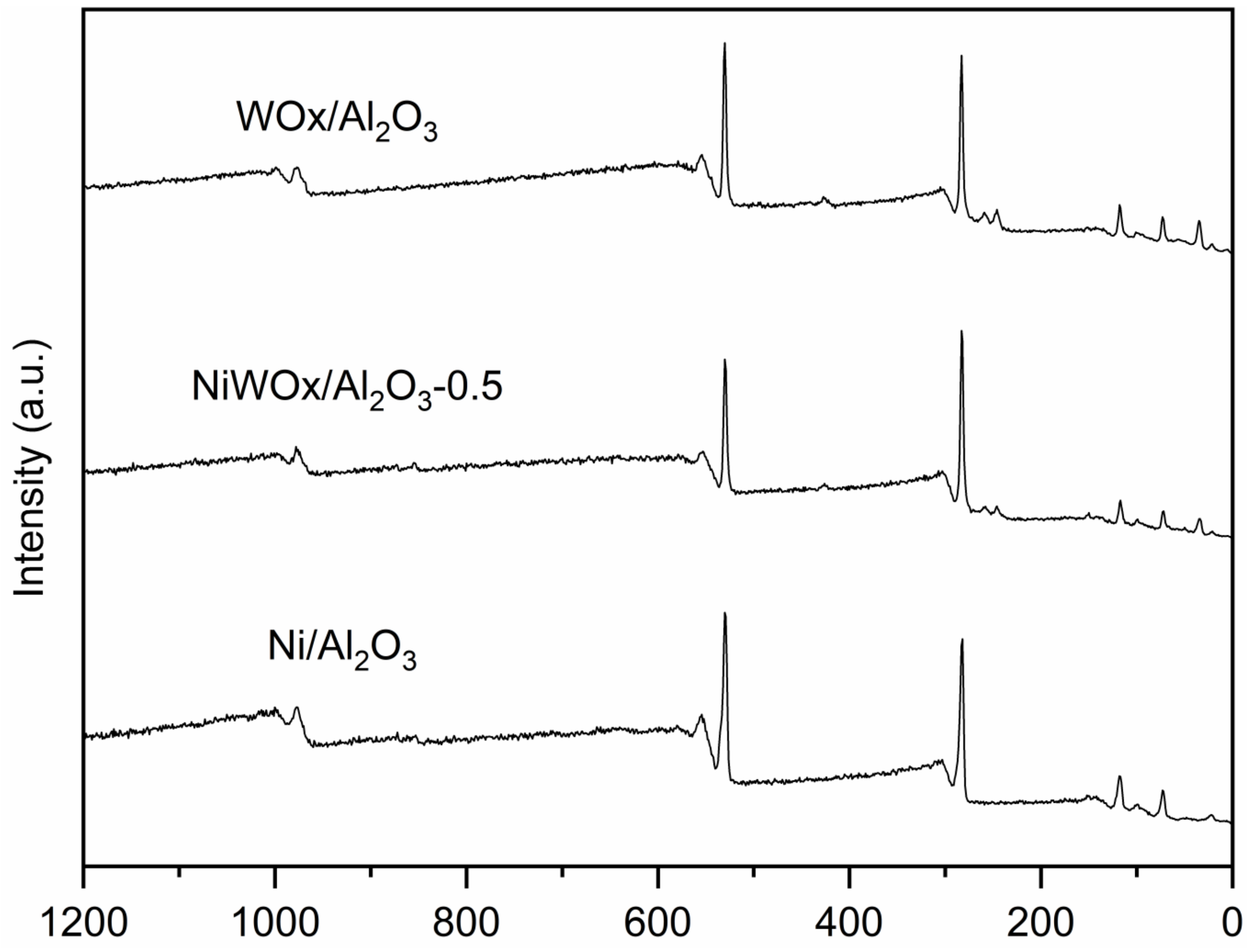

Supplementary Figure S3. The survey spectrum of Ni/Al_2O_3, $NiWOx/Al_2O_3$-0.5, and WOx/Al_2O_3.

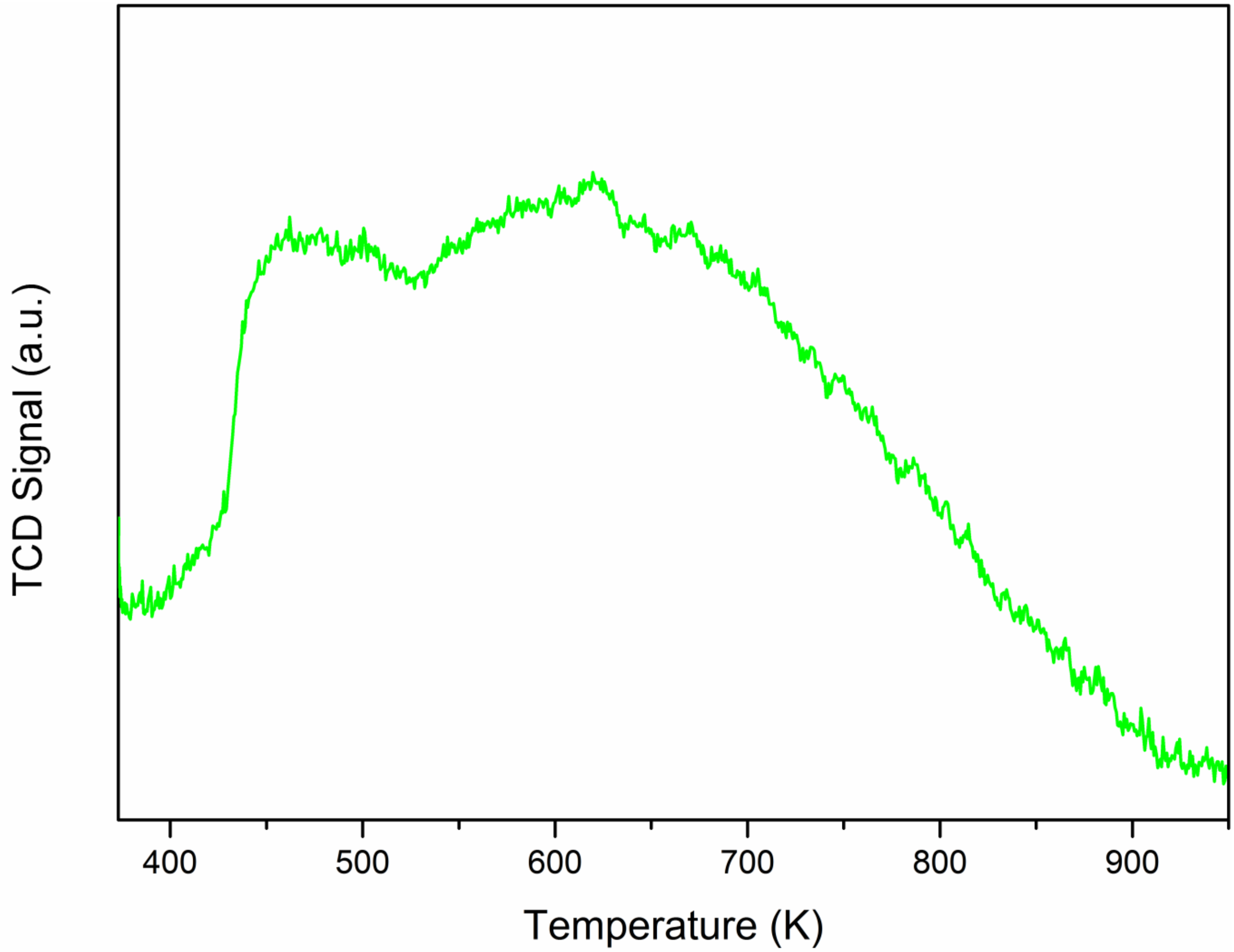

Supplementary Figure S4. The NH_3-TPD profiles of WOx/Al_2O_3.

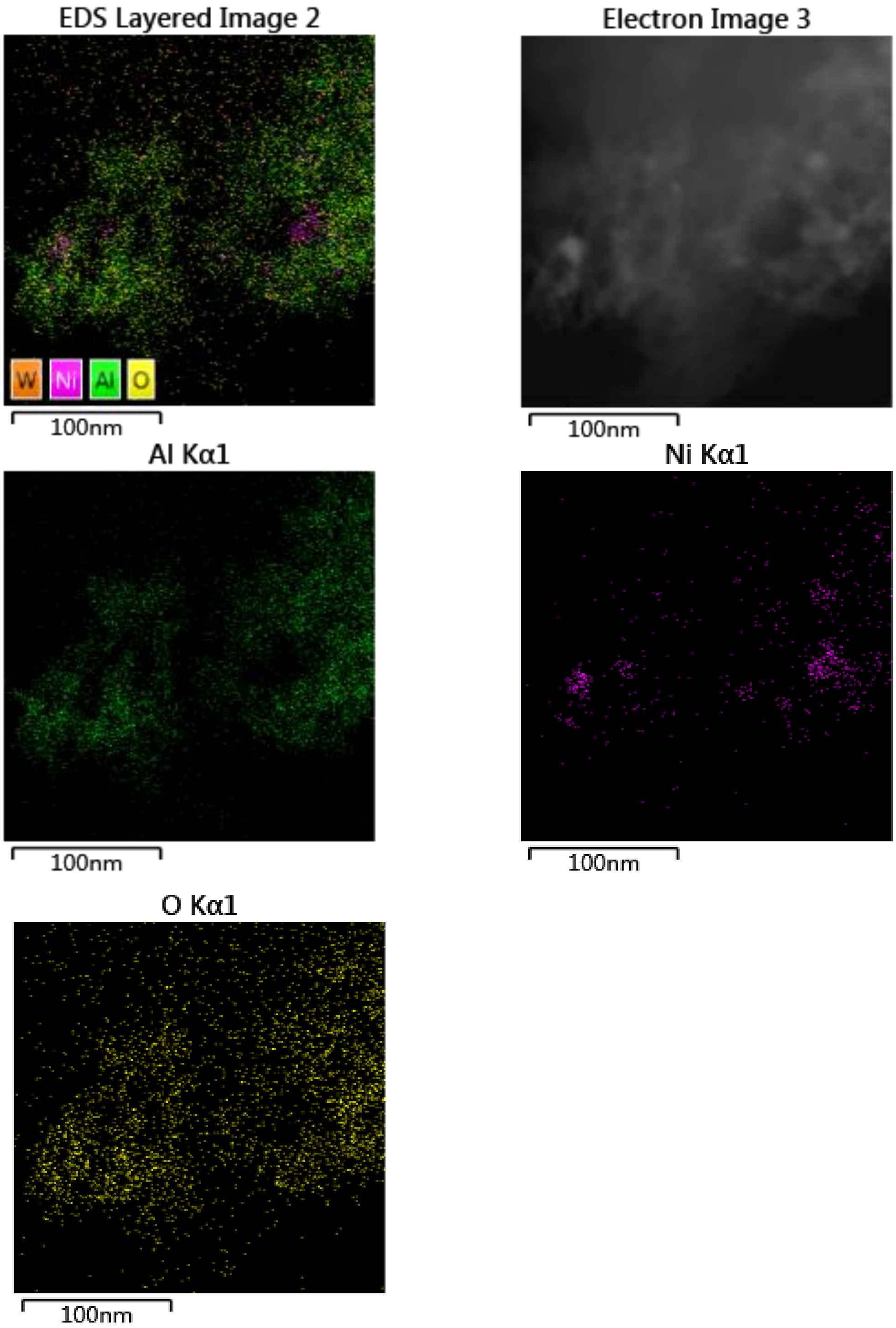

Supplementary Figure S5. The EDS mappings of Ni/Al_2O_3.

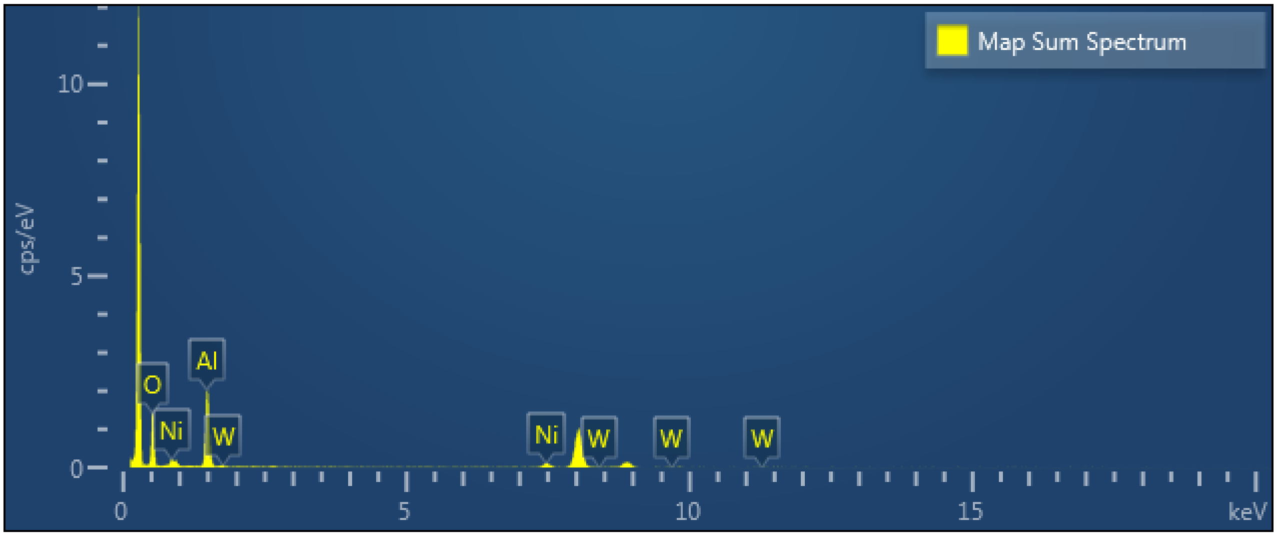

Supplementary Figure S6. The map sum spectrum for Ni/Al_2O_3.

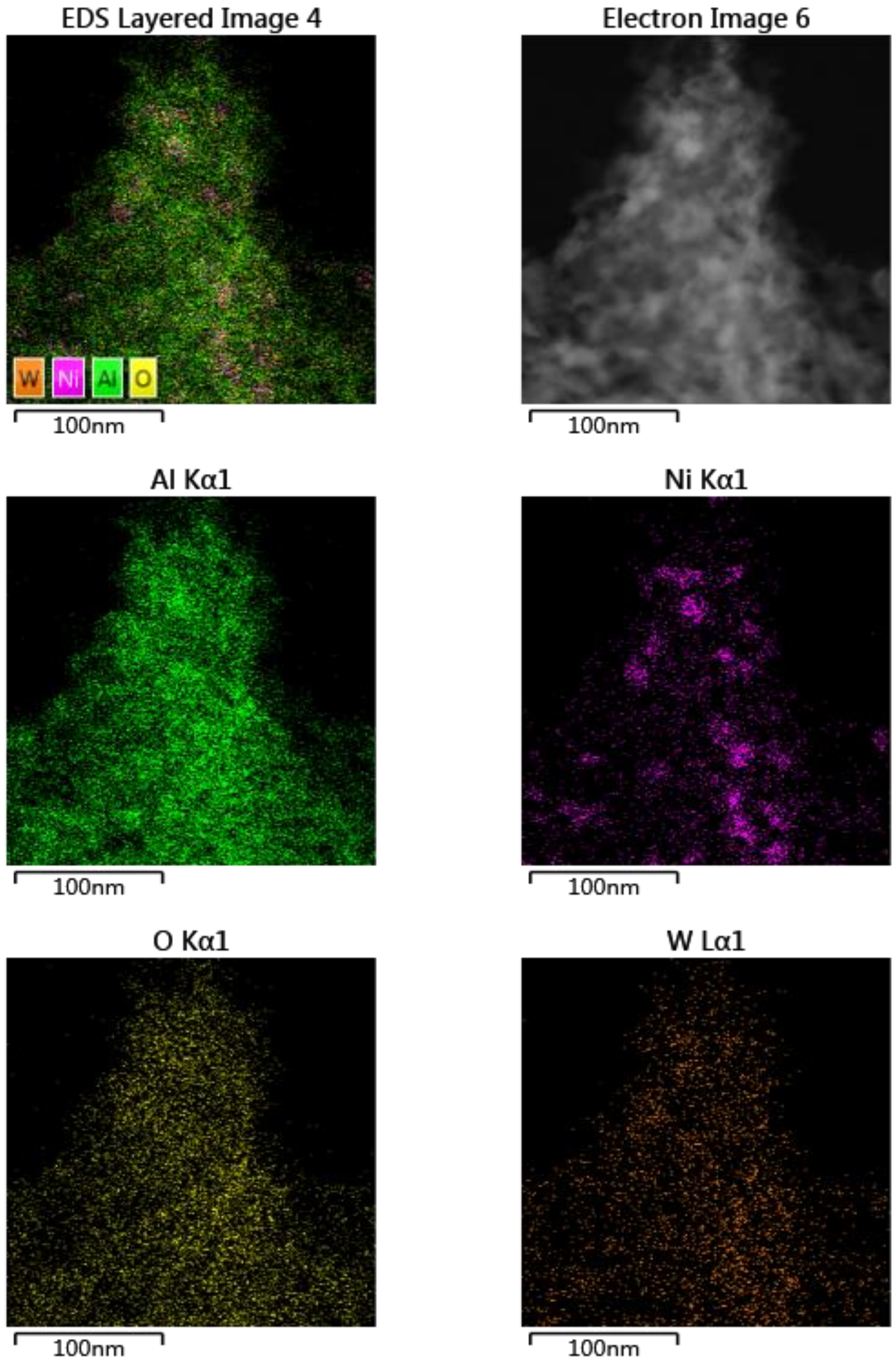

Supplementary Figure S7. The EDS mappings of Ni/Al_2O_3-0.5.

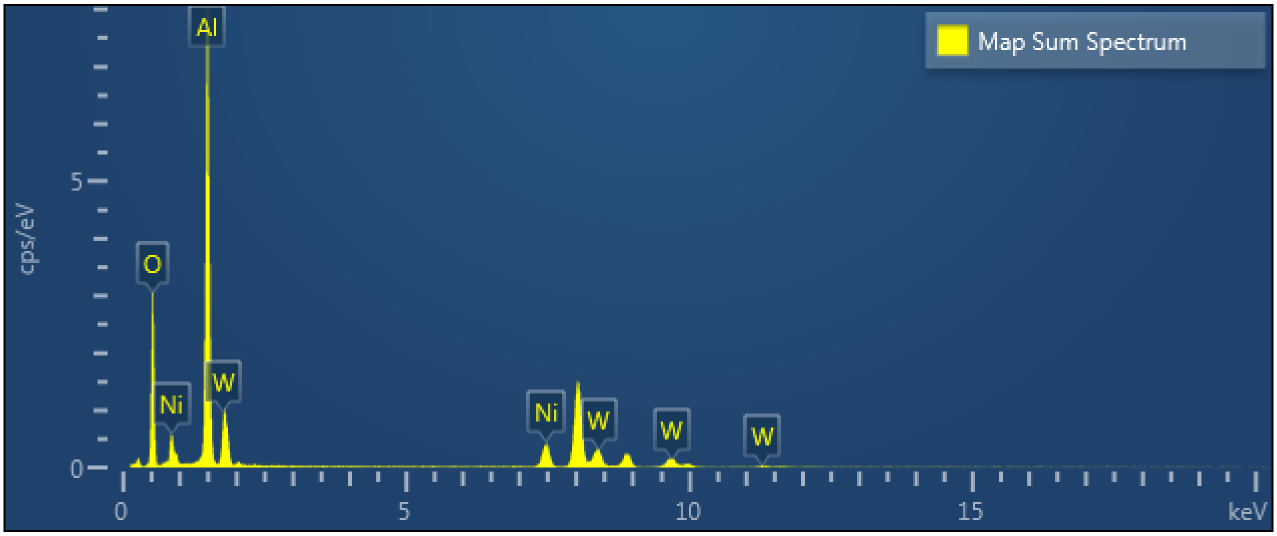

Supplementary Figure S8. The map sum spectrum for Ni/Al_2O_3-0.5.

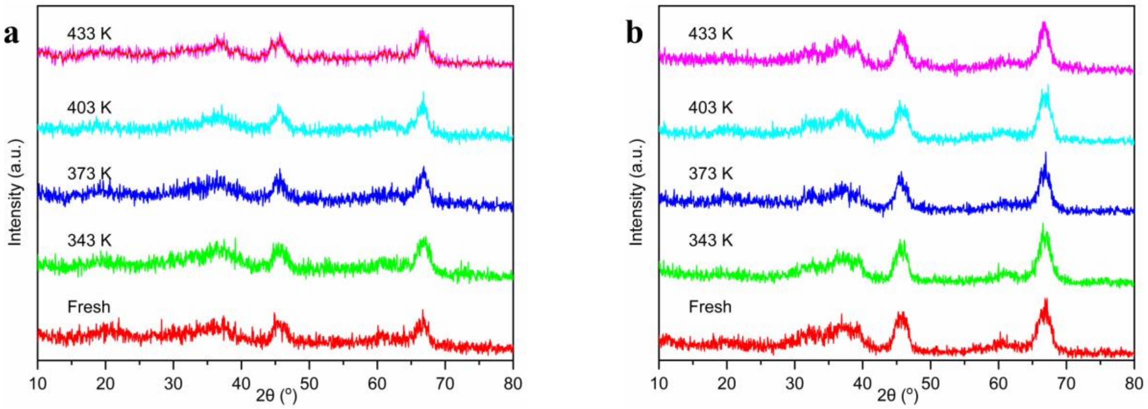

Supplementary Figure S9 The XRD patterns for NiWOx/Al_2O_3-0.5 and Al_2O_3 reacted at different temperatures.

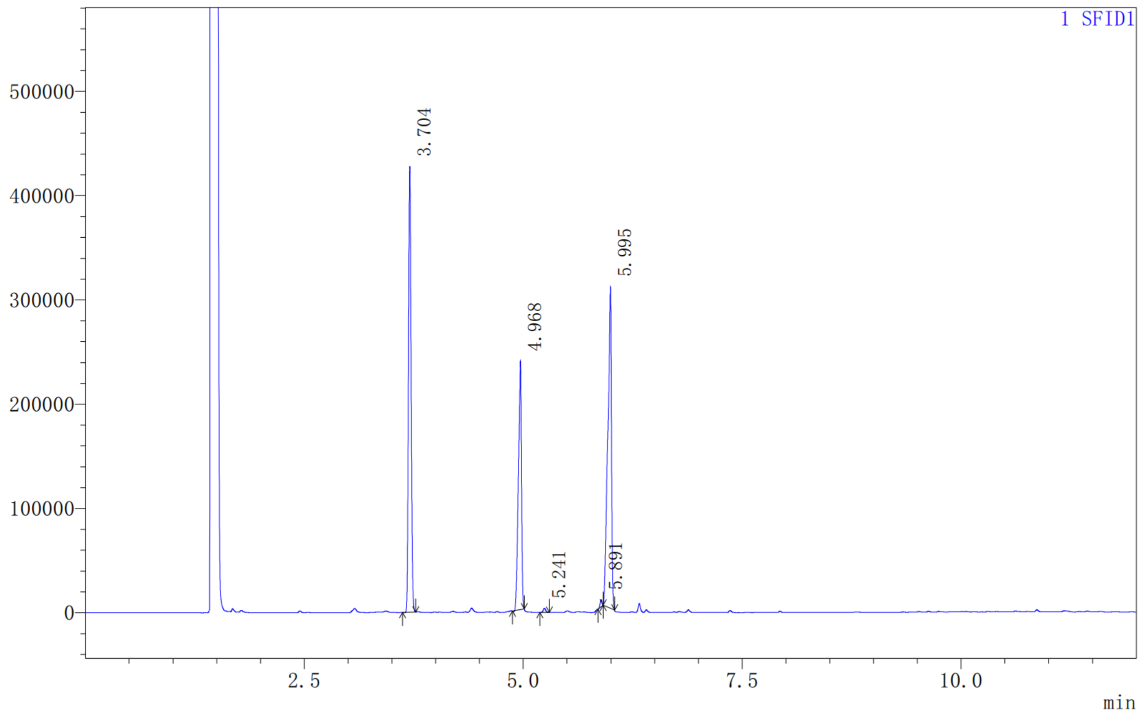

Supplementary Figure S10 Typical gas chromatogram for the hydrogenation of HMF. Reaction conditions: NiWOx/Al_2O_3-0.5 (20.0 mg), HMF solution (2.00 g, HMF: 1 mmol), H_2 (4 MPa), 393 K , 2 h. (t = 3.704 min, internal standard (n-decane); t = 4.968 min, HHD; t = 5.241 min, HCPO; t = 5.891 min, BHMF; t = 5.995 min, HMF; t = 6.999 min, 2,5-bis(hydroxymethyl)tetrahydrofuran; t = 7.272 min, 1,2,5-hexanetriol)

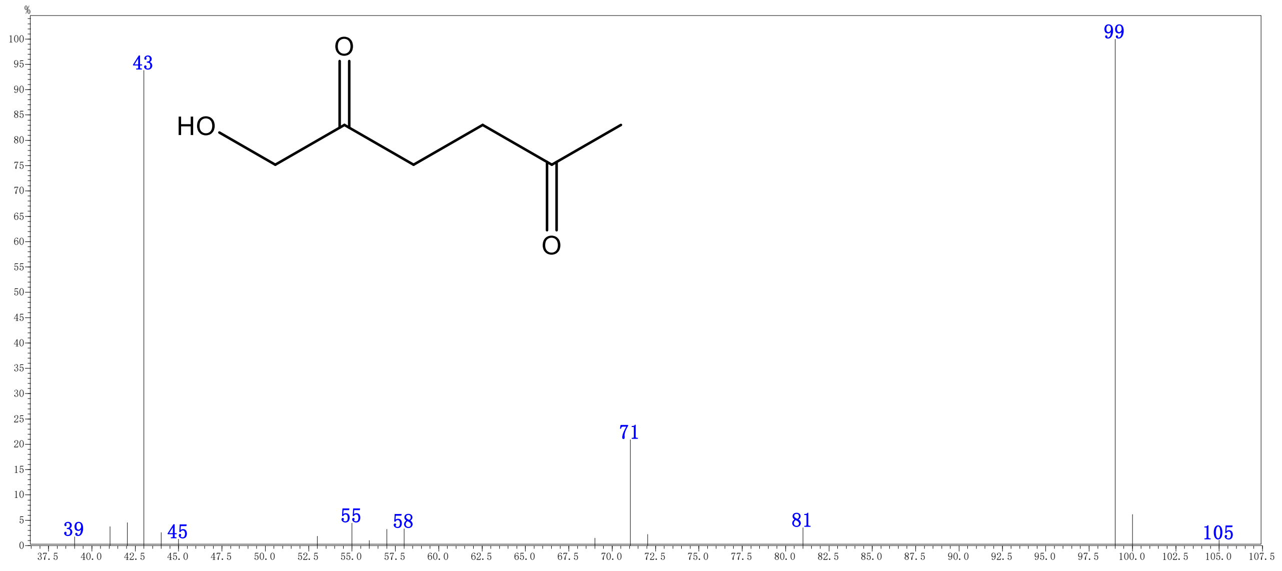

Supplementary Figure S11. The MS spectrum of HHD.

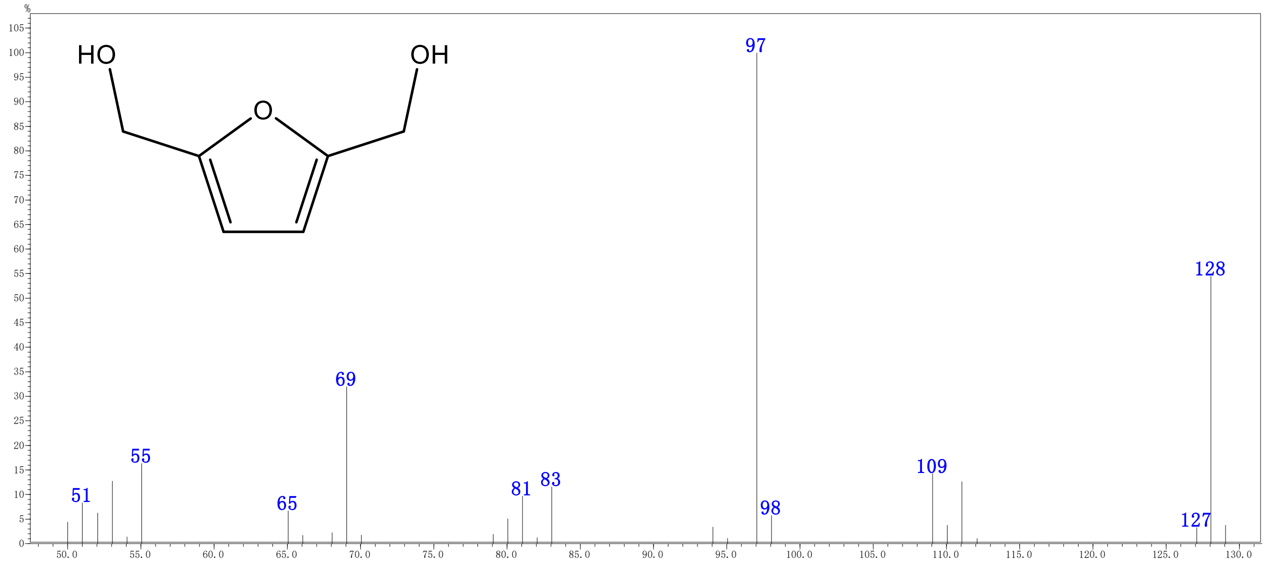

Supplementary Figure S12. The MS spectrum of BHMF.

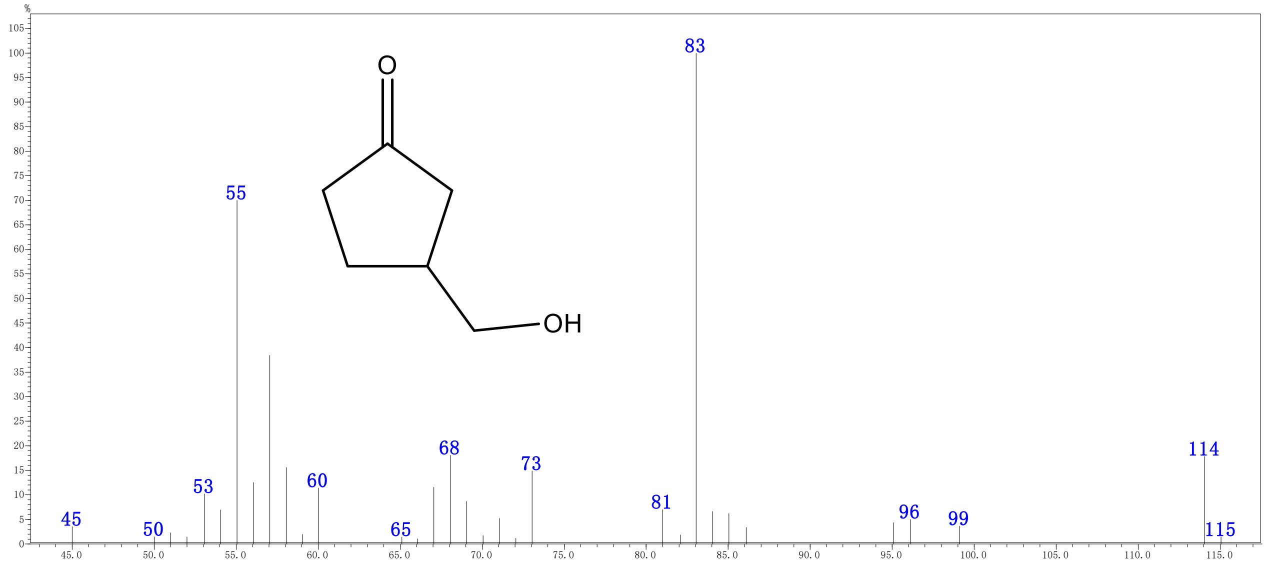

Supplementary Figure S13. The MS spectrum of HCPO.

2 Supplementary Tables

Supplementary Table S1 The corresponding peaks and H_2 consumption amount for H_2-TPR.

Entry	Catalysts	Reduction peaks	
		T (K)	H_2 (mL·g^{-1})
1	Ni/Al_2O_3	585-680	2.39
		680-893	22.89
2	$NiWOx/Al_2O_3$-0.5	547-615	0.84
		615-661	1.30
		661-876	15.39
3	WOx/Al_2O_3-0.5	657-792	0.45

Supplementary Table S2 The percentage of each element in Ni/Al_2O_3 and $NiWOx/Al_2O_3$-0.5.

Entry	Catalyst	Content (wt%)			
		Ni	W	Al	O
1	Ni/Al_2O_3	8.18	0	46.08	45.74
2	$NiWOx/Al_2O_3$-0.5	7.22	12.19	39.15	41.44
